# Jigsaw Puzzle Inspired Patterning of Gas Diffusion Layers for Enhanced Water Management in Polymer Electrolyte Fuel Cells

**DOI:** 10.1002/advs.202507918

**Published:** 2025-09-03

**Authors:** Enes Muhammet Can, Masamichi Nishihara, Kazunari Sasaki, Stephen Matthew Lyth

**Affiliations:** ^1^ Mechanical Engineering Department Faculty of Engineering and Natural Sciences Bursa Technical University Bursa 16310 Türkiye; ^2^ Mechanical Engineering Department Faculty of Engineering and Architecture Kırşehir Ahi Evran University Kırşehir 40100 Türkiye; ^3^ Next‐Generation Fuel Cell Research Center (NEXT‐FC) Kyushu University 744 Motooka, Nishi‐ku Fukuoka 819‐0395 Japan; ^4^ International Research Center for Hydrogen Energy Kyushu University 744 Motooka, Nishi‐ku Fukuoka 819‐0395 Japan; ^5^ Department of Hydrogen Energy Systems Graduate School of Engineering Kyushu University 744 Motooka, Nishi‐ku Fukuoka 819‐0395 Japan; ^6^ Strathclyde Incubator for Green Hydrogen Technologies (SigH_2_t) Department of Chemical and Process Engineering University of Strathclyde Glasgow G1 1XL UK

**Keywords:** fuel cell, gas diffusion layer, hydrophobicity, microporous layer, water management

## Abstract

Under high current density operation, water generation at the cathode of polymer electrolyte fuel cells (PEFCs) floods the electrode, resulting in severe mass transport limitation and an associated voltage drop. Water management is thus of crucial importance in improving the overall performance of fuel cell systems. Gas diffusion layers (GDLs) with independent pathways for either gaseous oxygen or liquid water transport present a potential solution to this issue. Here a novel, simple, and scalable method is presented for inducing patterned hydrophobicity into GDLs. Hydrophilic GDLs are prepared by immersion of commercial hydrophobic GDLs in hydrogen peroxide. Circular disks are then punched from these using a precision die‐cutter. Meanwhile, an array of corresponding holes is punched into conventional hydrophobic GDLs. The hydrophilic disks are then pressed into the holes of the hydrophobic GDL and held in place via friction locking, analogous to completing a jigsaw puzzle. This Jigsaw Puzzle Inspired Patterning (JPIP) technique creates precisely patterned hydrophilic domains to act as dedicated water channels, with separate hydrophobic domains for unhindered gas transport. The use of JPIP GDLs dramatically improves fuel cell performance under high current density operation, with important implications for decarbonization via the hydrogen economy.

## Graphical abstract

A novel jigsaw‐inspired method introduces patterned hydrophilic domains into gas diffusion layers (GDLs) to enhance water management in polymer electrolyte fuel cells (PEFCs). This scalable technique improves high‐current performance by separating water and gas pathways, offering a promising advance for hydrogen‐based energy systems.

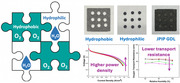

## 1 Introduction

**Figure 1:**
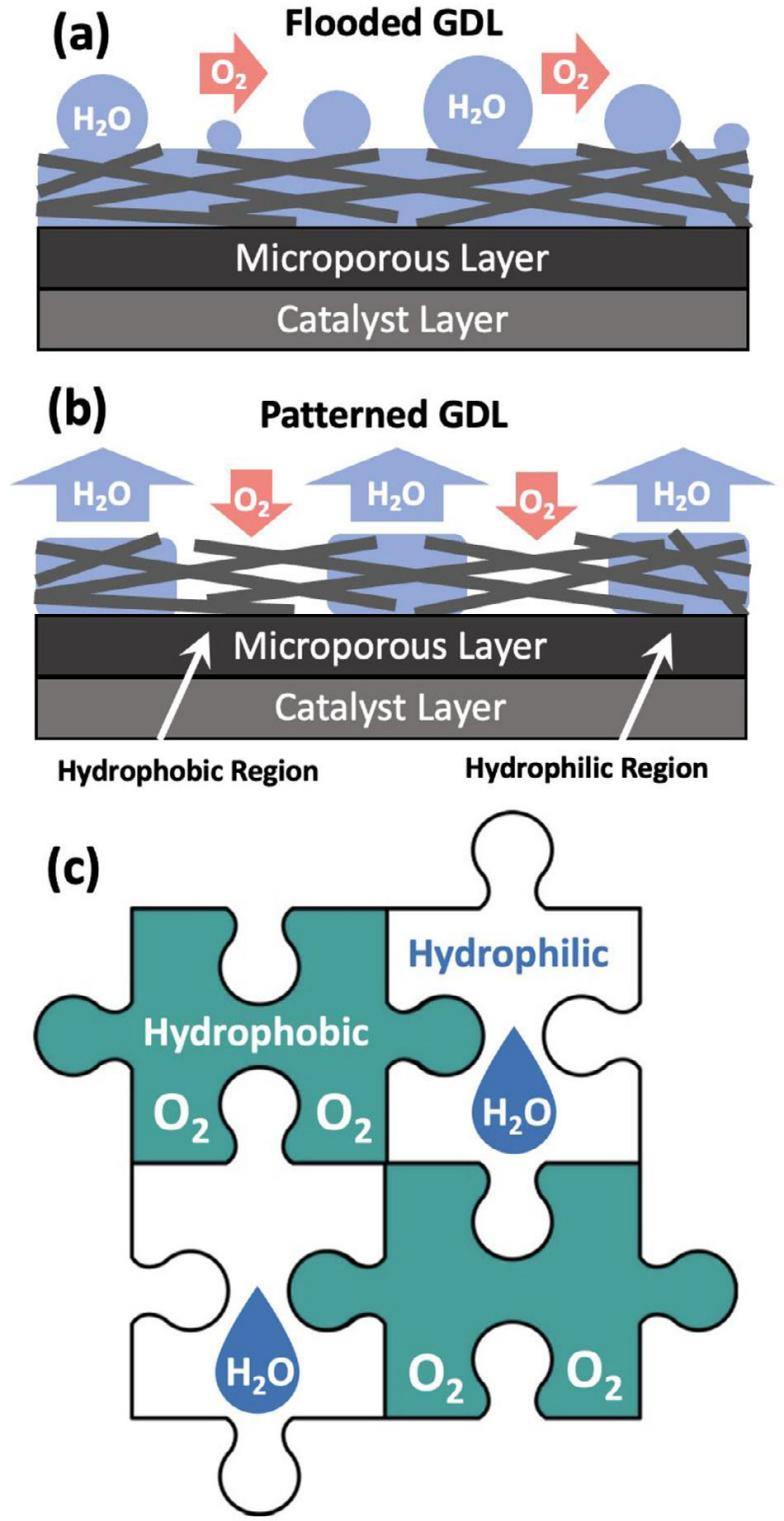
Schematic diagrams showing: a) flooding of a PEFC cathode using conventional GDLs under high current density operation, b) mitigation of flooding by the introduction of dedicated hydrophilic pathways for water transport, and hydrophobic pathways for oxygen transport; and c) the general concept of patterned wettability via Jigsaw Puzzle Inspired Patterning (JPIP).

Polymer electrolyte fuel cells (PEFCs) are a key energy conversion technology for the coming hydrogen economy. They can convert hydrogen to electricity with high power density and efficiency^[^ ^1^ ^]^ and their use in both stationery and mobile applications is on the rise.^[^ ^2^ ^]^ To further accelerate the usage of PEFCs in society there are several obstacles to be overcome, including cost, efficiency, and durability.^[^ ^3^, ^4^ ^]^ One important way to decrease the cost is to operate at high current density, thereby increasing the volumetric power density, resulting in reduced system volume and thus lower capital expenditure (CAPEX). However, as the operating current density of PEFCs increases, the rate of water generation in the cathode catalyst layer also increases correspondingly.^[^ ^5^ ^]^ If this water isn't removed from the cathode layer quickly enough, it can hinder oxygen transport to the electrocatalyst layer, resulting in a significant voltage drop, and thereby a decrease in system efficiency.^[^ ^6^, ^7^ ^]^ This phenomenon is called “flooding” (**Figure** **1**a,b) and is one of the main limiting factors in PEFC performance.

Currently, the main method for mitigating flooding in PEFCs is to use an appropriate gas diffusion layer (GDL) at the cathode. This critical PEFC component is a porous transport layer generally comprising carbon fiber or carbon paper and serves three main purposes: i) to transport gaseous oxygen from the flow field to the catalyst layer; ii) to transport the liquid water generated in the catalyst layer to the flow field; and iii) to provide electrical contact between the electrocatalyst layer and the bipolar plate. Water transport through porous solids (such as a GDL) is driven by capillary forces, which can be described by the Young‐Laplace equation (Equation 1).^[^ ^7^, ^8^ ^]^ In this relation, P_c_ is the capillary pressure, σ is surface tension, θ is the contact angle between water and the solid surface, and r is the effective pore radius:

(1) $$P_{C}=\frac{2\sigma \cos\theta }{r}$$

As such, the microstructure and wettability of the materials that make up a GDL have a significant impact on the behaviour of water in the system.^[^ ^9^, ^10^, ^11^ ^]^ In hydrophilic materials (i.e., where θ <90° and thus cos θ >0) positive capillary pressure favours water uptake which can lead to pore saturation (i.e., flooding) if not properly managed. Commercial GDLs for PEFCs are therefore coated with a hydrophobic agent such as polytetrafluoroethylene (PTFE). In hydrophobic materials (i.e., where θ >90° and thus cos θ <0) negative capillary pressure resists water intrusion into the larger pores, maintaining gas pathways.^[^ ^12^, ^13^, ^14^ ^]^ Meanwhile, capillary forces still allow liquid water intrusion into smaller pores (due to the inverse relationship between P_c_ and r), providing routes for water transport to the flow field. Furthermore, a microporous layer (MPL, comprising a thin coating of carbon black and a PTFE binder) is generally applied to the GDL.^[^ ^6^, ^15^, ^16^, ^17^, ^18^, ^19^ ^]^ This has smaller pores than the GDL, assisting with water transport, whilst also acting to decreases the contact resistance between the catalyst layer and the GDL.^[^ ^6^, ^20^, ^21^ ^]^ Although coating with PTFE and the addition of an MPL tackles water management to a certain degree thus is a delicate balance, and flooding remains a serious issue at higher current density. As such, further advances in GDL / MPL composition and design are required to enable higher current density operation.

The maximum power density (or peak power density) commonly observed in fuel cell polarisation curves can be directly attributed to the onset of flooding (or alternatively membrane dry‐out under different operating conditions).^[^ ^22^, ^23^ ^]^ As such the current density at maximum power density (I_MP_) is a simple and convenient parameter to quantify the effect of flooding on PEFC performance. One method to enable water management at even higher current densities is to create patterned GDLs with macroscopic regions of differing hydrophilicity and hydrophobicity (Figure 1b,c). This creates capillary pressure gradients, meaning that water will tend to wick from relatively hydrophobic regions toward relatively hydrophilic regions. This is analogous to a passive capillary pump in microfluidic devices, presenting a potentially powerful and controllable way to further enhance water management in PEFCs. However, considering the complex microstructure of a GDL (and MPL), creating patterned configurations is not straightforward.

Several approaches to creating patterned wettability have been proposed. One common method is the application of polymer coatings to selected regions of the GDL or MPL. For example, Koresawa et al. coated stripes or dots of PTFE (∼1 mm feature size) onto untreated GDLs with the aid of vacuum suction, resulting in improved oxygen diffusion, although no in situ cell tests were reported.^[^ ^24^ ^]^ Similarly, Sakaida et al. painted Nafion onto hydrophobic GDLs using a brush to create relatively hydrophilic regions, resulting in a modest improvement in I_MP_ from ≈0.55 to ≈0.65 mA cm^−2^.^[^ ^25^ ^]^ Meanwhile, we previously took a similar approach, painting hydrophobic PTFE onto untreated GDLs through stainless‐steel masks in three different patterns, decreasing the oxygen transport resistance and increasing the I_MP_ from ≈1.00 to 1.12 mA cm^−2^ as well as providing insight into how the positioning of hydrophobic/hydrophilic regions relative to the outlet affects performance.^[^ ^26^ ^]^ Despite the relative success of coating GDLs with polymers of different surface energy, several issues remain, including bleeding of the applied coating (limiting the precision); compatibility between the selected polymer and the underlying GDL; and the water solubility of some coatings selected in the above studies. Meanwhile, the use of electronically insulating polymers increases the ohmic resistance of the GDL.^[^ ^11^ ^]^

Addressing some of the above issues, Forner‐Cuenca et al. employed radiation‐induced graft copolymerization of fluorinated ethlyene propylene to create GDLs with precisely patterned hydrophilic and hydrophobic stripes, reporting that narrower hydrophilic domains increased the required capillary pressure for water infiltration.^[^ ^27^ ^]^ Later in a series of three papers, they i) investigated the effect of contact angle by varying the polymer type and loading; ii) used ex‐operando neutron and synchrotron imaging to visualise the water distribution within the GDL; and iii) confirmed that the cell performance was correspondingly improved (with I_MP_ increasing from ≈1.25 to 1.75 A cm^−2^).^[^ ^28^, ^29^, ^30^ ^]^ The same group then developed pore network models to investigate this system, predicting that Toray GDLs with hydrophilic domains of 500 µm in width would demonstrate optimal performance.^[^ ^31^, ^32^ ^]^ Overall, this is an interesting method to induce patterning in GDLs, although large‐scale application of radiation grafting techniques is expected to add significant extra time and cost and to commercial GDL manufacturing processes.

Other groups have also focused on controlling the wettability of MPLs. Bae et al. used inkjet printing to deposit MPLs with different contact angles onto GDLs in a variety of patterns, increasing the I_MP_ from 1.2 to 1.3 A cm^−2^.^[^ ^33^ ^]^ Chun et al., sprayed MPLs with different hydrophilic and hydrophobic polymer components onto GDLs through a grating mask to achieve stripes of ≈0.5 mm in width resulting in better water transport (but did not present any fuel cell data).^[^ ^34^ ^]^ Another Wang et al. created composite MPLs via incomplete blending of polyvinylidene difluoride (PVDF) and Nafion / titania coated carbon blacks, increasing the I_MP_ from ≈0.4 to 0.5 A cm^−2^.^[^ ^35^ ^]^ These methods appear promising, but the reported data show only modest improvements to fuel cell performance to date.

Another relatively common method that has been used to induce patterning in GDLs is laser treatment, which can create hydrophilic regions via local heating effects. Many of these studies have involved perforating GDLs and MPLs with hydrophilic holes. For example, Wang et al. created water channels by perforating GDLs using a laser, with diameters varying from 80 to 200 µm, resulting in an improvement in I_MP_ from ≈1.8 to 2.0 A cm^−2^.^[^ ^36^ ^]^ Lin et al. performed a similar procedure, drilling hydrophilic channels in GDLs to achieve an improvement in I_MP_ from ≈2.2 to 3.5 A cm^−2^.^[^ ^37^ ^]^ Meanwhile, Wen et al. prepared a patterned water‐draining GDL by laser drilling entirely through a hydrophobic layer to an underlying hydrophilic layer, impressively increasing I_MP_ from 2.0 to 3.6 A cm^−2^.^[^ ^38^ ^]^ Other groups have created hydrophilic tracks or grooves in the MPL using laser treatment. For example, Iglesia et al. fabricated hydrophilic grooves in GDLs via laser micro‐machining resulting in an improvement in I_MP_ from ≈2.0 to 2.2 A cm^−2^.^[^ ^39^ ^]^ Similarly, Zang et al. etched ≈0.5 mm hydrophilic tracks with different spacings on a hydrophobic GDL with a UV laser, reportedly improving the I_MP_ from 0.9 to 1.35 mA cm^−2^.^[^ ^40^ ^]^ Most recently, Ding et al. fabricated GDLs with graduated hydrophilic grooves via laser processing, increasing I_MP_ from ≈3.0 to 3.7 A cm^−2^.^[^ ^41^ ^]^ Some of the above laser patterning techniques result in an impressive increase in PEFC performance, but the additional cost and complexity of such a process should be considered, as well as the potential safety issues and ecological impact of the decomposition of fluorinated polymers under laser irradiation.

An alternative method of introducing patterned structures is by stamping or pressing with a template to create indentations in the MPL. Wang et al. created micro‐grooves in MPLs as an artifact of the screen‐printing deposition process, resulting in an increase in I_MP_ from ≈0.7 to 0.8 mA cm^−2^.^[^ ^42^ ^]^ Similarly, Zhou et al. created grooves by impressing a nylon sieve into an MPL under pressure, reporting an increase in I_MP_ from 1.15 to 1.27 A cm^−2^.^[^ ^43^ ^]^ Although interesting, this method appears to result in only modest improvement in performance.

Finally, in an alternative technique, Wang et al. coated GDLs with either hydrophobic or hydrophilic MPLs by blending carbon black with PTFE or polyamide resin, respectively. They cut the resulting GDLs into 1 mm strips and reassembled them in an alternating configuration within a PEFC assembly, observing significantly improved performance under cold‐start operation (but without presenting fuel cell polarisation curves).^[^ ^44^ ^]^ The above studies have all introduced patterned wettability into the GDL or MPL of fuel cells and observed improved performance. They are all broadly similar in methodology, and present similar mechanisms for the improved performance, namely that water wicks preferentially into the hydrophilic regions, leaving the hydrophobic regions available for oxygen transport.

Here, we propose a new and simple method to fabricate GDLs precisely patterned with distinct hydrophilic and hydrophobic regions. This method involves i) punching circular holes in a predetermined pattern in a conventional hydrophobic carbon fiber GDL; ii) production of a hydrophilic GDL via chemical oxidation of a conventional hydrophobic GDL in hydrogen peroxide; iii) punching disks from the hydrophilic GDL; and iv) inserting the hydrophilic disks into the holes of the hydrophobic GDL. We term this method Jigsaw Puzzle Inspired Patterning (JPIP, Figure 1c). Differently from previously published studies, in this case the underlying GDL is patterned whilst the coated MPL is homogeneously hydrophobic. Furthermore, we employ chemical treatment of GDLs to induce hydrophilicity without compromising the electronic conductivity. This is a simple, reproducible, and scalable way to create GDLs with precisely patterned regions of tailored hydrophilicity for water management in various electrochemical applications.

## 2 Methodology

### 2.1 Materials

All chemicals were used as received without further purification. Uncoated non‐woven graphitized carbon fiber GDLs were used in this work (SGL GDL‐29AA, SGL Carbon, Germany). Hydrogen peroxide (30%), and methyl cellulose 4000 were purchased from Wako, Japan. Triton X‐100 was obtained from Sigma Aldrich. Polytetrafluoroethylene (PTFE, Teflon 30B, 60 wt.%) was purchased from Polyscience, Inc. Acetylene carbon black (100% compressed) was purchased from Strem Chemicals, Inc.

#### 2.1.1 Hydrophilic GDL Treatment

In this work, chemical functionalization is selected as a suitable method for increasing the hydrophilicity of GDLs without compromising the electronic conductivity compared with alternative methods such as polymer coating or plasma functionalization.^[^ ^45^, ^46^, ^47^, ^48^, ^49^ ^]^ Hydrogen peroxide was selected as a relatively safe, mild oxidant allowing a greater degree of control over the carbon oxidation process compared with harsher alternatives such as nitric or sulfuric acid which can alter the microstructure of carbon fibers due to gasification. A 4 × 4 cm GDL (SGL‐GDL‐29AA) was placed in a 300 mL round‐bottom flask, immersed in 150 mL of 30% H_2_O_2_ solution, and heated to 90 °C under reflux using an oil bath. The duration of treatment was varied between 12, 24, and 48 h to control the oxygen content.

### 2.2 GDL Punching and Reassembly

**Figure 2:**
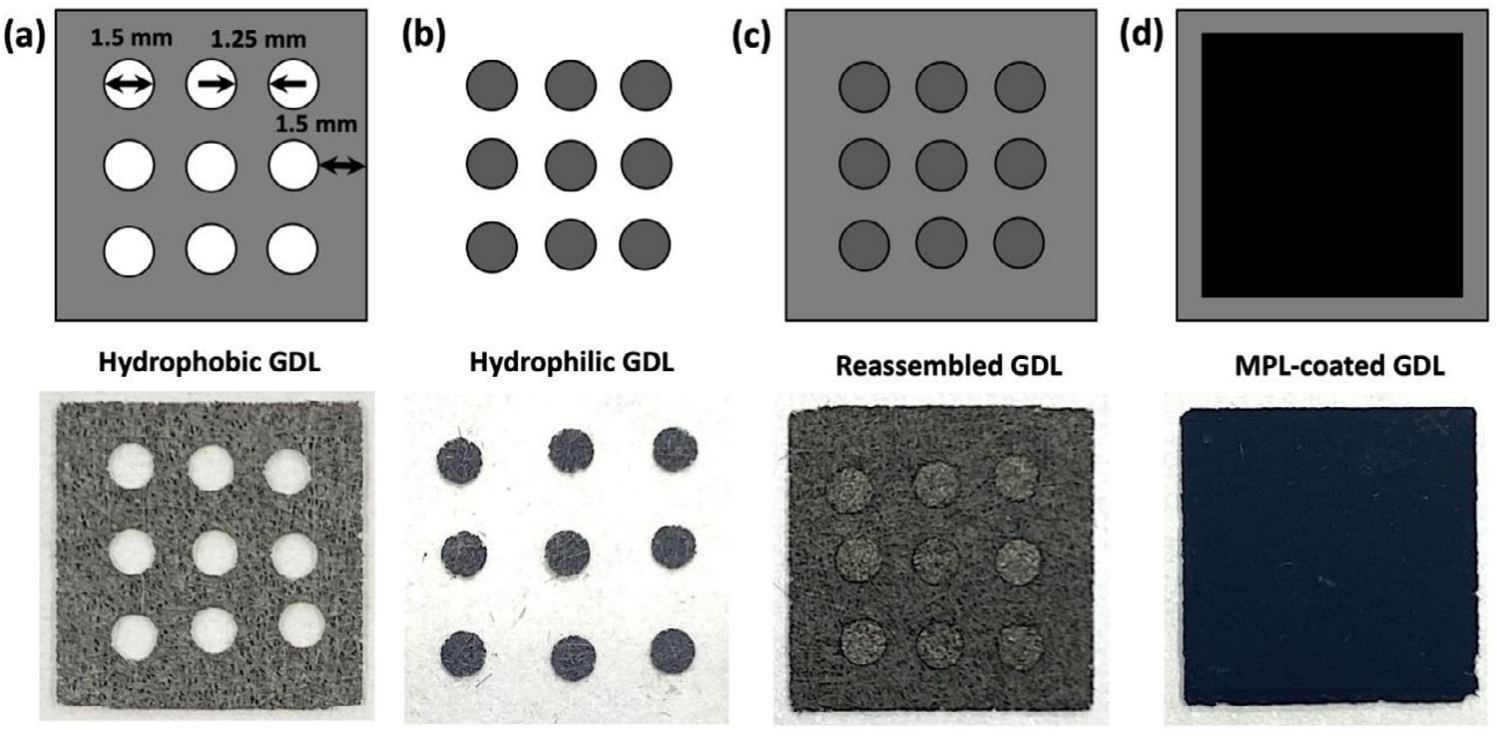
Schematic diagram (top row) and photographs (bottom row) of the Jigsaw Puzzle Inspired Patterning (JPIP) method. a) Holes are punched in a conventional hydrophobic GDL. b) Disks are punched from a chemically oxidized hydrophilic GDL. c) The hydrophilic disks are gently press‐fitted into the holes of the hydrophobic GDL. d) A conventional MPL is then coated over the entire surface of the reassembled hybrid GDL.

**Figure 3:**
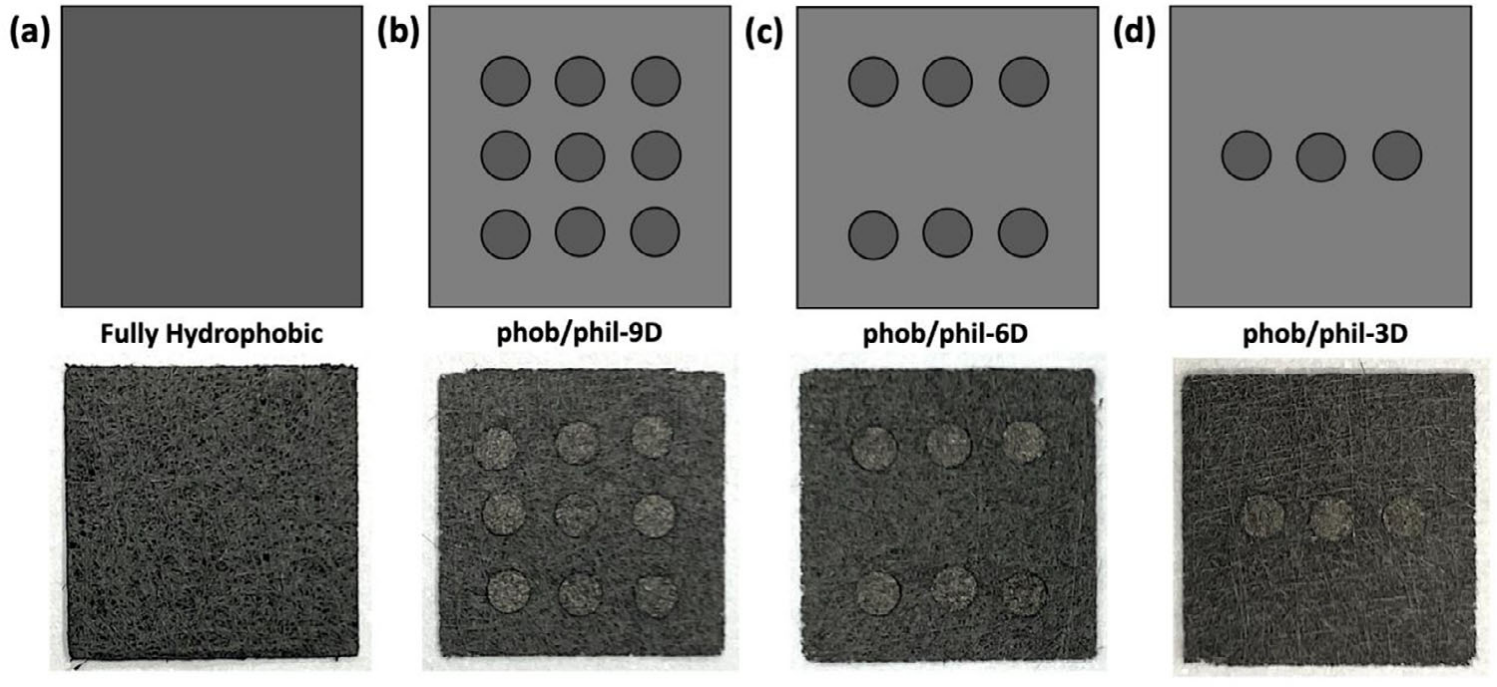
Schematic diagram (top row) and photographs (bottom row) of the different geometric configurations investigated using the JPIP method: a) fully hydrophobic; b) nine hydrophilic disks (phob/phil‐9D); c) six hydrophilic disks (phob/phil‐6D); d) three hydrophilic disks (phob/phil‐3D).

A handheld precision electrode punch (Nogami‐ken Co., Ltd., Japan, ± 4 µm tolerence) was then used to cut 1.5 mm diameter disks from as‐received SGL‐GDL‐29AA which was cut into 1 × 1 cm squares (**Figure** **2**a). The same punch was used to cut 1.5 mm diameter disks from the peroxide‐treated hydrophilic GDLs (Figure 2b). Then, the hydrophilic disks were carefully press‐fitted into the corresponding holes in the hydrophobic GDL, where they were securely held in place via friction locking (Figure 2c). Several geometrical configurations were prepared with either 3, 6, or 9 punched holes (**Figure** **3**). The distance between the closest edges of the circles was 1.25 mm, and the distance from the edges of the circles to the edge of the GDL was 1.5 mm (Figure 2a). The diameter was selected considering the results of previous studies,^[^ ^24^ ^]^ as well as in consideration of the physical handling of the small parts.

### 2.3 MPL Coating

After combining the punched hydrophobic GDLs with the hydrophilic disks to form the hybrid JPIP GDLs, an MPL was applied to the surface (Figure 2d). This was performed after reassembly (rather than before punching the hydrophobic and hydrophilic GDLs) to simplify the process, improve the mechanical integrity of the resulting GDL via partial bonding, and to ensure electrical connectivity across the entirety of the GDL. To prepare the MPL slurry, 2 g of acetylene carbon black, 0.5 g of methyl cellulose 4000, 0.1 mL of Triton X‐100, and 13 mL of deionized water were placed in a 100 mL polypropylene beaker. The inclusion of methyl cellulose 4000 and Triton X‐100 improves the dispersibility and rheological attributes of the slurry during processing, as well as increasing the porosity of the final MPL. The resulting mixture was dispersed using a homogenizer (Thinky Mixer AR‐100) for 15 min at 2000 rpm. Then, 0.572 mL of 60 wt.% PTFE solution was added, as a hydrophobic binder. The mixture was homogenized again for 15 min at 2000 rpm to create an MPL slurry. The target PTFE content in the final MPL was 20 wt.%.

GDLs (4 cm × 4 cm) were placed on a glass plate, and a 75 µm‐thick stainless‐steel frame with a 3 cm × 3 cm window was carefully clamped on top. The MPL slurry was then coated directly onto the GDLs using a doctor blade and dried at 70 °C for 1 h. The MPL‐coated GDLs were then heat‐treated at 380 °C in air for 30 min, to remove the methyl cellulose and Triton X‐100, and to sinter the PTFE.^[^ ^8^, ^19^ ^]^ The thickness of the resulting MPLs was highly uniform at 40 ± 3 µm (as measured by Mitutoyo Digital Micrometer).

To quantify the porosity of the resulting MPLs, the slurry was also coated directly onto a glass plate using the same methodology to form free‐standing MPLs. The porosity was then estimated from the thickness, area, mass, and density of the different components, using the following equation^[^ ^8^, ^19^ ^]^:

(2) $$\phi =1-\frac{V_{S}}{V_{MPL}}=1-\frac{m_{MPL}\cdot \left(\omega_{c}/\rho_{c}+\omega_{B}/\rho_{B}\right)}{d_{MPL}\cdot A}$$

where ∅ is the porosity of the MPL; ***V***_***S***_ is the volume of the solid components (i.e., PTFE and acetylene carbon black); ***V***_***MPL***_ is the geometric volume of the MPL; ***m***_***MPL***_is the mass of the MPL; ***ω**_c_* is the mass fraction of the acetylene carbon black (0.80); ***ω**_B_* is the mass fraction of the PTFE binder (0.20); ***ρ***_c_ is the density of the acetylene carbon black (1.75 g cm^−3^); ***ρ***_B_ is the density of the PTFE binder (1.75 g cm^−3^); ***d***_***MPL***_ is the thickness of the MPL; and ***A*** is the area of the MPL (1 cm^2^). Three different free‐standing MPLs were prepared using stainless steel frames of 50, 75, and 100 µm thickness, resulting in MPL thicknesses of 30, 40, and 55 µm, respectively. The three corresponding porosities were 77.2, 77.2, and 77.4 %, respectively, confirming the high reproducibility of this doctor blade technique. MPLs were coated onto the JPIP GDLs in an identical manner, but with a smaller 1 × 1 cm window in the stainless‐steel frame.

### 2.4 Characterization

Scanning electron microscopy (SEM, Hitachi SEM‐SU9000) was used to observe the structure and morphology of the GDLs. X‐ray photoelectron spectroscopy (XPS, PHI 5000 Versa probe (II) ULVAC) was used to determine the elemental composition near the GDL surfaces. Water contact angle (WCA) measurements of GDLs were also performed (DMs‐401, Kyowa Interface Science Co., Ltd, Japan) with 1 µL droplets, with each sample measured 10 times and the average WCA quoted.

### 2.5 MEA Preparation

To prepare catalyst ink, Pt/C (46.8 wt_Pt_.%, TEC10V50E, Tanaka, Japan), 5 wt.% Nafion solution (Wako, Japan), deionized water, and super‐dehydrated ethanol (Wako, Japan) were first homogenized in a 100 mL polypropylene beaker at 500 rpm for 30 min (Thinky Mixer AR‐100). The resulting catalyst ink was then coated onto a 100 µm thick‐ PTFE sheet using an auto film applicator (Tester Sangyou Co., LTD PI‐1210), and dried in an oven at 65 °C for 1 h. Subsequently, the catalyst layer was transferred from the PTFE sheet to a Nafion 212 membrane by hot pressing at 140 °C and 0.3 kN for 180 s (Sinto Digital Press CYPT‐10). The catalyst loading was 0.3 mg_Pt_ cm^−2^ at both the anode and cathode, and the active area was 1 cm^2^.

### 2.6 Single Cell Characterization

A single cell was fabricated according to guidelines published by the New Energy and Industrial Technology Development Organization (NEDO) in Japan. The active area was 1 cm^2^, with parallel flow channels. The gas flows for regular current‐voltage (I‐V) performance curves were set to 0.332 L min^−1^ air and 0.139 L min^−1^ hydrogen for cathode and anode, respectively (counter flow conditions). Cell temperature, gas flow rate, and humidity were controlled by an automated fuel cell test station (AUTOPEM‐CVZ01, Toyo Corporation, Japan). An electrochemical interface impedance analyzer (Solartron SI‐1287) was used for I‐V performance measurements. All measurements were performed at a scan rate of 5 mA s^−1^ at 95% relative humidity and 80 °C cell temperature. Prior to performance tests, each cell was conditioned at 0.6 V for 16 h.

The total oxygen transport resistance (*R_T_*, s m^−1^​) was determined based on the limiting current density at different values of relative humidity (80, 85, 90, 95, and 100 %), using the following equation,^[^ ^8^, ^19^, ^50^ ^]^ where F is the Faraday constant (96 485 C mol^−1^), *P_O2_* is the oxygen partial pressure (Pa), *R* is the gas constant (8.314 J mol^−1^K^−1^), T is the cell temperature (in K) and *i_lim_* is the limiting current density at 0.2 V:

(3) $$R_{T}=\frac{4F\cdot P_{O_{2}}}{i_{\lim}\cdot R\cdot T}$$

Hydrogen was supplied to the anode at 1 L min^−1^ and a mixture of nitrogen (98 vol.%) and oxygen (2 vol.%) was supplied to the cathode also at 1 L min^−1^. Prior to oxygen transport resistance measurements, the cell conditions were stabilized for 15 min.

## 3 Results and Discussion

### 3.1 Hydrophilic GDL Treatment

**Figure 4:**
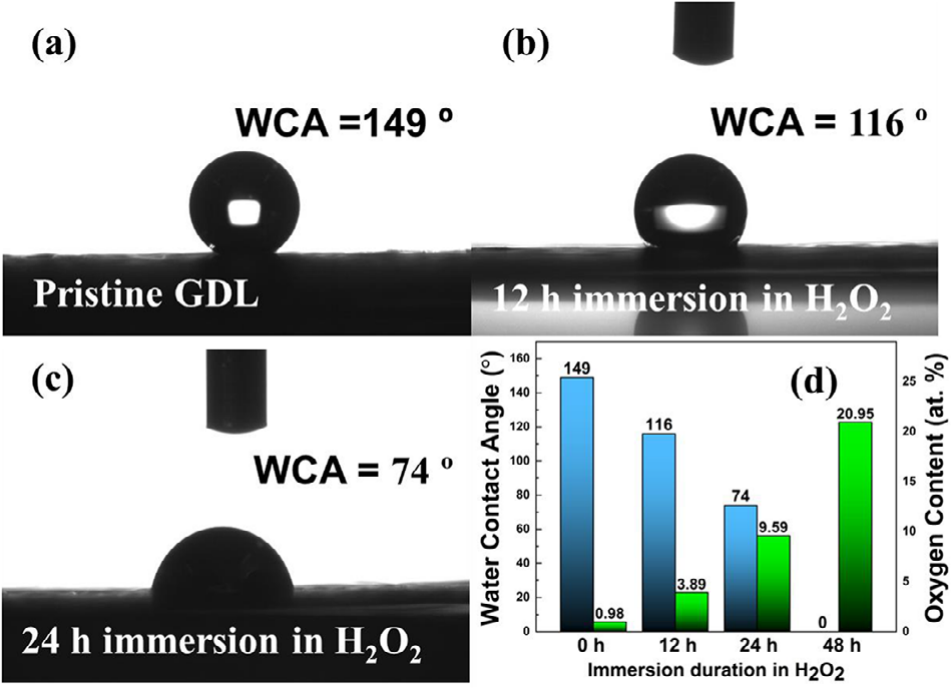
Water contact angles of SGL 29 AA GDLs a) before, and after immersion in hydrogen peroxide for b) 12, and c) 24 h. After 48 h of immersion, the WCA was 0°. d) Graph plotting changes in water contact angle and oxygen content as the duration of the hydrogen peroxide treatment increases.

First, the method of surface functionalisation of the GDLs was investigated. Uncoated GDLs were immersed in hydrogen peroxide for varying amounts of time to make them hydrophilic to different degrees. The resulting oxygen content near the surface of the GDL was quantified using XPS (**Figure** **4**d; Figures S1–S4, Supporting Information) and found to be proportional to the duration of immersion in hydrogen peroxide, increasing from ≈1.0 at.% for the pristine GDL, to 21.0 at.% after 48 h of oxidative treatment. The clear trend in oxygen content confirms that the degree of surface functionalization can be precisely tailored using this simple technique. Other methods using, e.g., nitric acid are much harsher and can potentially lead to uncontrolled rapid oxidation and gasification of carbon, leading to undesirable changes in microstructure.

In general, oxygen‐containing functional groups dramatically impact the hydrophilicity of carbon materials. Therefore, the water contact angles (WCA) of peroxide‐treated GDLs were measured using the sessile drop technique (Figure 4a–c). In close agreement with the changes in oxygen content, there is a clear decrease in contact angle as the peroxide immersion time increases (Figure 4d). The pristine GDL has a WCA of 149° which decreases to 116° after immersion in H_2_O_2_ for 12 h, and 74 ° after immersion for 24 h. After immersion for 48 h, the water droplet wets the surface and is adsorbed into the GDL, and thus the WCA is assumed to be 0°. Again, these measurements confirm that the degree of GDL hydrophilicity can be precisely controlled by immersion in hydrogen peroxide for varying amounts of time.

### 3.2 Fully Hydrophobic JPIP GDLs

Next, for benchmarking, the effect of punching and reassembly of GDLs on the microstructure and performance was investigated without introducing patterned wettability and compared with a conventional unmodified MPL‐coated GDL (SGL 29AA). This was achieved by employing the JPIP method as usual with 6 punched discs (Figure 3c), but in this special case the reinserted disks were left untreated and thus remained fully hydrophobic. This means that the whole area of the GDL was nominally homogeneous in terms of wettability and that any change in performance could be attributed inherently to the JPIP method itself rather than any local changes in hydrophilicity.

The precision die‐cut hydrophilic disks pressed into the corresponding spaces in the hydrophobic GDL remained securely in place throughout the whole manufacturing process including handling, MPL‐coating, cell assembly, and cell disassembly. As such, it can be assumed that the disks are held in place via friction locking, similar to the case of press‐fitted mechanical parts in industrial settings. This is then supplemented by the application and subsequent heat treatment of the MPL, resulting in partial bonding between the reinserted disks and the GDL matrix, further enhancing the mechanical integrity of the interface and reducing the risk of dislodgment during processing.

**Figure 5:**
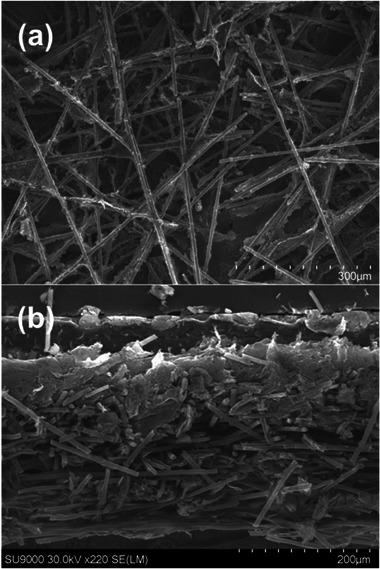
Representative SEM images of a JPIP GDL interface after re‐assembly: a) surface (before coating with an MPL), and b) cross‐sectional image (after MPL coating).

The surface of the reassembled GDLs was observed by SEM before coating with the MPL (**Figure** **5**a; Figure S5, Supporting Information). The point at which the host GDL and the reinserted disk meet is almost indistinguishable from the rest of the GDL, but on close inspection individual severed carbon fibers can be observed in all the images. The fibers of the different components overlap in many cases, suggesting that the disks are physically held in place via friction locking. The extremely close fit of the discs in the host GDL is attributed to the high precision of the punch, which has a nominal tolerance of just ± 4 µm, which is extremely small compared with the diameter of the disks. Indeed, the reassembled GDLs are remarkably robust during physical handling at this stage of the process, with no hint of the re‐inserted disks shifting or coming loose. After coating with the MPL, the location of the reinserted disks is completely indistinguishable from the rest of the GDL (Figure S6, Supporting Information). A cross‐sectional image of the MPL‐coated GDL (Figure 5b) again reveals severed but overlapping carbon fibers at the interface indicating that the disks are held in place by friction locking, with a continuous MPL at the top of the image. Overall, these results indicate that the JPIP method can be achieved with high precision and that the pieces are friction locked in position.

**Figure 6:**
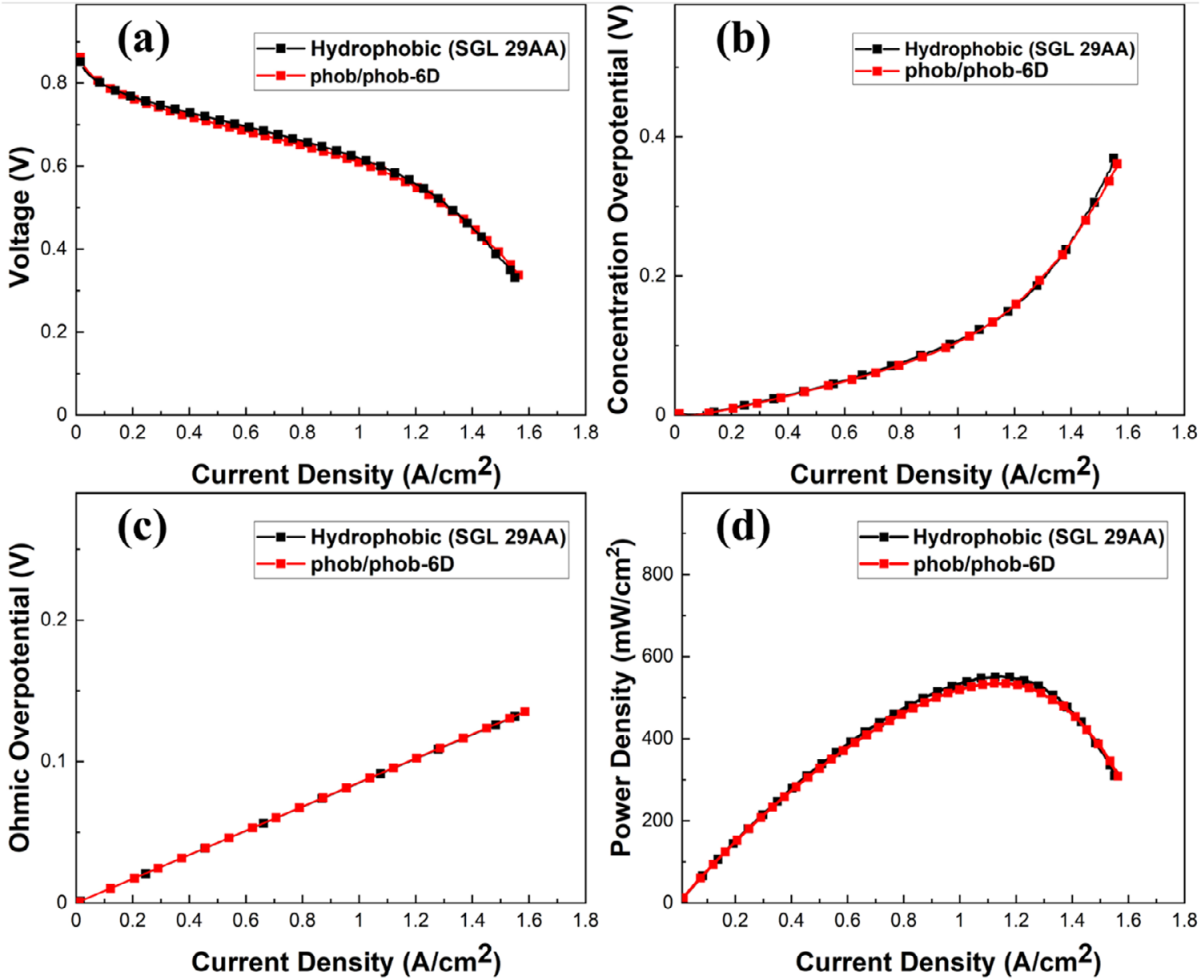
IR‐free polarization curves of PEFCs fabricated using the fully hydrophobic benchmark JPIP GDL (phob/phob‐6D), compared with a conventional hydrophobic GDL (SGL 29AA). a) *I*–*V* polarisation curves; b) concentration overpotential; c) ohmic overpotential; d) power density curves. All measurements were conducted at 80 °C, under 95 % relative humidity, with a hydrogen flow rate of 139 mL min^−1^ and an air flow rate of 332 mL min^−1^.

The resulting benchmark JPIP GDLs were then assembled into fuel cells as outlined in the methods section and labelled *phob/phob‐6D*. The resulting IR‐free polarization curves are shown in **Figure** **6**a. It is immediately clear that there is a negligible difference between the use of the conventional intact hydrophobic GDL, and the punched and reassembled JPIP GDL. This confirms that the process of punching and reassembling GDLs does not inherently affect the initial fuel cell performance. Figure 6b–d show the concentration overpotential, ohmic overpotential, and power density of PEFCs using the phob/phob‐6D GDLs, and the oxygen transport resistance is shown in Figure S7 (Supporting Information). These results all provide further confirmation that the JPIP method has no significant inherent positive or negative effect on mass transport through the GLD or electronic conductivity of the GDL (without also varying the hydrophobicity as shown in the following sections).

### 3.3 Varying the Hydrophilic/Hydrophobic Area Ratio

**Figure 7:**
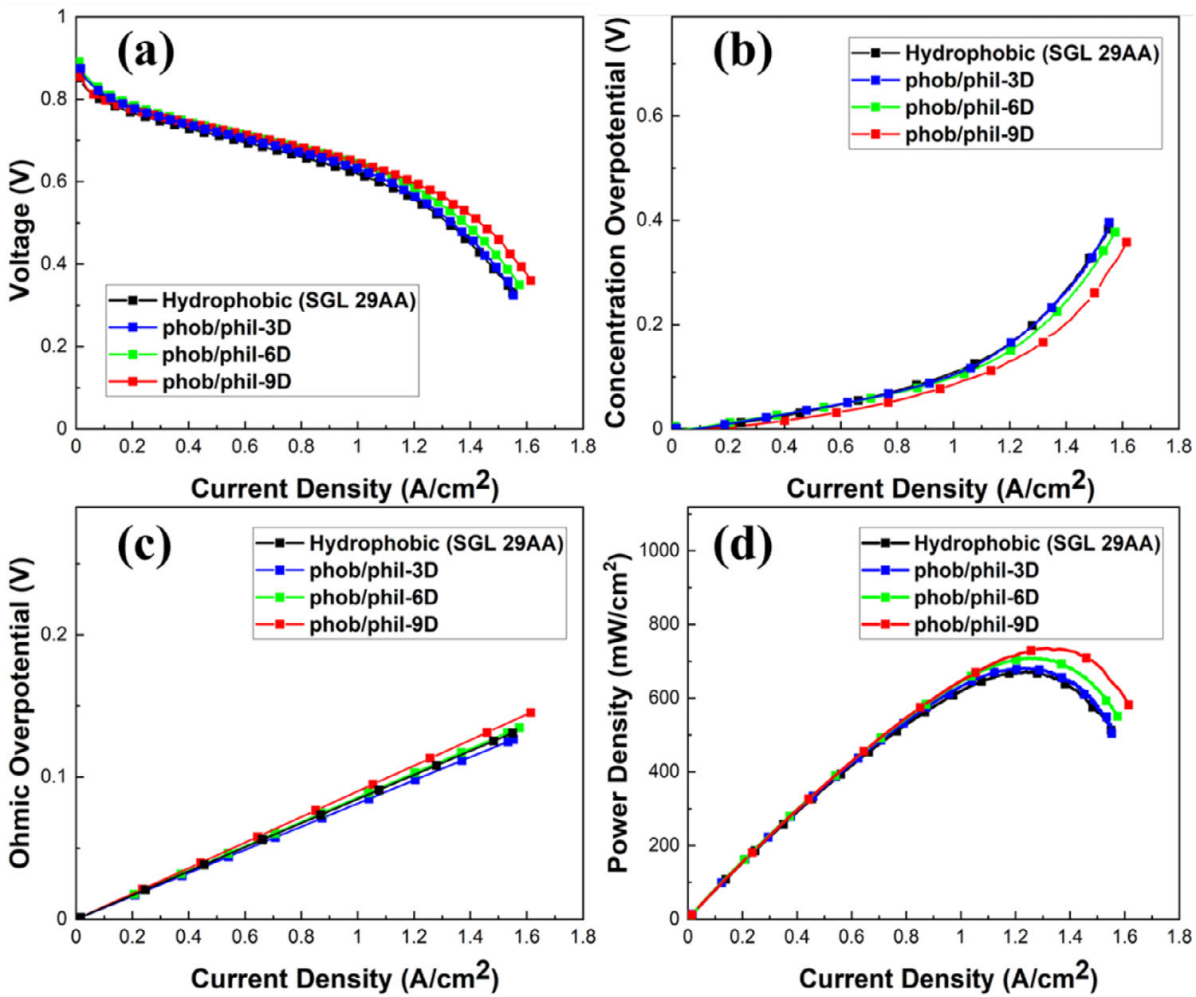
Performance of PEFCs fabricated using JPIP GDLs fabricated with either 3, 6, or 9 hydrophilic disks (phob/phil‐3D, phob/phil‐6D, and phob/phil‐9D, respectively) compared with a conventional MPL‐coated GDL (SGL 29AA). a) IR‐free polarization curves; b) concentration overpotential; c) ohmic overpotential; and d) power density curves.

Next, hydrophilic disks (12 h immersion in H_2_O_2_, WCA = 116°) were inserted into the hydrophobic host GDL. The effect of changing the relative areas of the hydrophobic and hydrophilic domains was investigated by varying the number of punched discs in the GDL (as shown in Figure 3). Three hydrophilic disks (phob/phil‐3D) corresponds to an area ratio of 5.3%; six disks (phob/phil‐6D) to a ratio of 10.6%, and nine disks (phob/phil‐9D) to 15.9%, relative to the total area of the GDL. The resulting polarization curves are compared with a conventional hydrophobic GDL (SGL 29AA) in **Figure** **7**.

In this case, a clear improvement in PEFC performance is observed after the introduction of hydrophilic domains using the JPIP method, in clear contrast with the benchmark GDLs. The positive effect on performance increases as the number of hydrophilic domains increases. In the I‐V curves (Figure 7a), the limiting current density for the fully hydrophobic reference GDL is 1.55 A cm^−2^ at 0.33 V. Increasing the hydrophilic area ratio to 5.3% (3 disks) does not significantly change the *I*–*V* performance. However, increasing further to 10.6% (6 disks) significantly increases the limiting current density to 1.57 A cm^−2^ at 0.35 V. Increasing the hydrophilic area ratio even further to 15.9% (9 disks) further increases the limiting current density to 1.62 A cm^−2^ at 0.36 V.

The concentration overvoltage (or mass transfer overvoltage) has been well‐established to be linked to water flooding in PEFCs under high current density operation, as confirmed by, e.g., in situ pressure drop measurements^[^ ^19^, ^51^, ^52^, ^53^, ^54^, ^55^, ^56^ ^]^ Figure 7b confirms that the improvement in PEFC performance can be mainly attributed to a significant decrease in concentration overpotential as the relative area of the patterned hydrophilic GDL increases. This strongly indicates that the provision of dedicated hydrophilic channels in this system can minimise the effects of flooding.

Meanwhile, the ohmic overpotential is a measure of the conductivity of the system (including both ionic and electronic components). In this case, the membranes and electrocatalyst layers are identical in all cells, so any differences can be attributed to changes in the electronic conductivity of the GDLs. There is a very slight increase in ohmic overpotential as the number of hydrophilic domains increases, but not enough to negate the benefits of water management (Figure 7c). This slight increase may be due to increased resistance at the surface of the peroxide‐treated GDLs due to the additional oxygen functional groups, since no increase was observed in the benchmark JPIP GDLs.

The fuel cell power density curves also reflect the overall improvement in performance (Figure 7d). The peak power density of the hydrophobic reference GDL is 669 mW cm^−2^ (I_MP_ ≈1.23 A cm^−2^). This increases significantly to 681, 710, and 735 mW cm^−2^ as the number of hydrophobic disks the GDL increases, corresponding to I_MP_ values of 1.25, 1.27, and 1.34 A cm^−2^, respectively. This further confirms the above trends and strongly suggests that the onset of flooding is shifted to higher current density due to the incorporation of hydrophilic channels in the GDL.

Furthermore, the oxygen transport resistance was directly measured between 80 and 100 % relative humidity to gain direct insight into mass transport within the patterned GDLs during operation (Figure S8, Supporting Information). In a similar trend to the above results, there is a significant decrease in oxygen transport resistance as the number of hydrophilic disks in the JPIP GDL increases. This provides further evidence that flooding can mitigated by using this patterning technique to incorporate dedicated hydrophilic channels for water transport.

The clear trend observed above is attributed to the higher proportion of hydrophilic areas available for effective water wicking through the GDL. In the case of phob/phil‐3D, the limited number of hydrophilic water channels saturates relatively quickly as the current density increases, and the water quickly penetrates into the surrounding hydrophobic regions, increasing the oxygen transport resistance (i.e., flooding). Increasing the number of hydrophilic channels (i.e., for phob/phil‐6D and phob/phil‐9D) results in a higher current density being reached before saturation with water occurs. It was found that increasing the number of hydrophilic disks in the JPIP GDL even further (e.g., to 12) compromised the structural integrity of the GDL during punching. As such, determining the optimal ratio and geometry of hydrophilic and hydrophobic domains will be an important topic for future studies employing the JPIP method. Overall, the above results unequivocally confirm the beneficial effect of employing JPIP GDLs for water management in PEFCs, as well as clarifying that the ratio of hydrophobic to hydrophilic domains plays an important role.

### 3.4 Varying the Degree of Hydrophilicity

**Figure 8:**
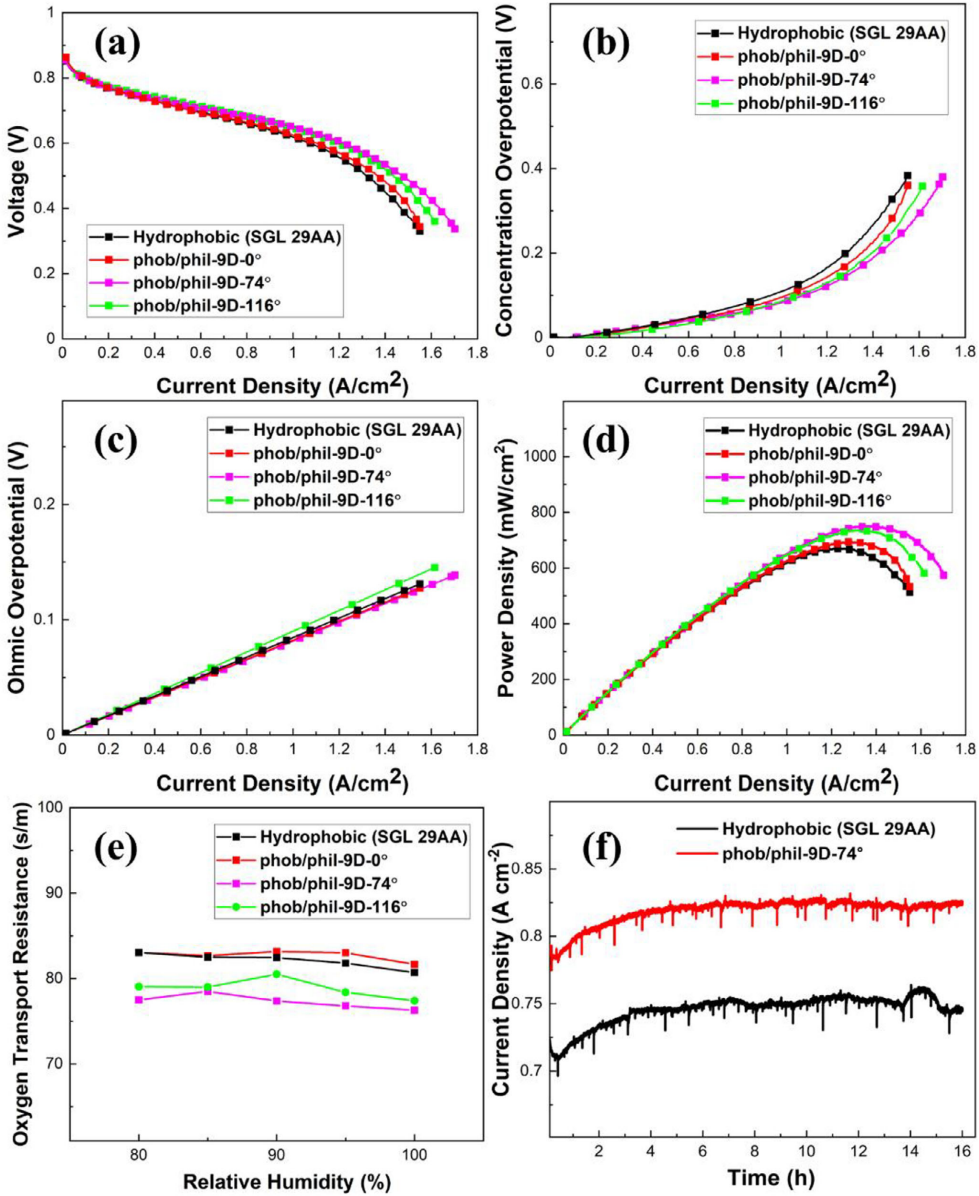
Performance of PEFCs fabricated using JPIP GDLs fabricated with hydrophilic disks of varying water contact angle (116°, 74°, and 0°, respectively) compared with a conventional MPL‐coated GDL (SGL 29AA): a) IR‐free polarization curves; b) concentration overpotential; c) ohmic overpotential; d) power density curves; e) oxygen transport resistance; and f) stability tests for the best‐performing sample (phob/phil‐9D‐74°).

To gain further insights into the effect of JPIP GDLs on fuel cell performance and water management, the degree of hydrophilicity was also investigated (**Figure** **8**). GDLs with different water contact angles (116°, 74°, and 0°) were prepared by immersing hydrophobic GDLs in hydrogen peroxide solution for different periods of time, as previously described in Figure 4. Disks were then punched from these hydrophilic GDLs and pressed into the hydrophobic GDL matrix in the 9D configuration as outlined in the previous section.

In all three cases, the *I*–*V* characteristics are improved significantly by incorporating the hydrophilic disks (Figure 8a). The conventional hydrophobic reference GDL has a limiting current density of 1.55 A cm^−2^ at 0.33 V. This increases to 1.62 A cm^−2^ at 0.36 V for the least hydrophilic disks (phob/phil‐9D‐116). A further improvement to 1.71 A cm^−2^ at 0.34 V is then observed for the disks with intermediate hydrophilicity (phob/phil‐9D‐74). However, for the most hydrophilic discs (phob/phil‐9D‐0), a slight decrease in limiting current density to 1.55 A cm^−2^ at 0.34 V is observed, and the performance is comparable to the reference GDL.

As in the previous section, the concentration overpotential (Figure 8b) confirms that the trends in performance can largely be attributed to changes in mass transport within the cell, with phob/phil‐9D‐74 clearly displaying the lowest concentration overpotential. Meanwhile, only minor changes in the ohmic overpotential are observed, with no clear trend (Figure 8c). This again confirms that the JPIP method has minimal impact on the electronic conductivity of the GDLs.

Meanwhile, the peak power density for the reference GDL is 669 mW cm^−2^ (I_MP_ ∼1.23 A cm^−2^), increasing to 735 mW cm^−2^ for phob/phil‐9D‐116 (I_MP_ ≈1.35 A cm^−2^), further increasing to 751 mW cm^−2^ for phob/phil‐9D‐74 (I_MP_ ≈1.38 A cm^−2^), and slightly decreasing again to 695 mW cm^−2^ for phob/phil‐9D‐0 (I_MP_ ≈1.28 A cm^−2^). This further confirms that the onset of flooding is shifted to higher current density due to the incorporation of hydrophilic channels in the GDL.

Furthermore, the oxygen transport resistance was measured to determine trends in mass transport within the patterned GDLs (Figure 8e). In agreement with the polarization curves, the oxygen transport resistance is highest in the case of the conventional hydrophobic GDL, indicating that flooding impedes oxygen transport to the electrocatalyst. The values decrease significantly for phob/phil‐9D‐116, and decrease even further for phob/phil‐9D‐74. Finally, for phob/phil‐9D‐0 the oxygen transport resistance increases again, becoming comparable with the conventional hydrophobic GDL. These results follow the same trends noted above and confirm that they can be attributed to enhanced oxygen transport via improved water management.

Finally, durability tests were carried out on the best‐performing GDL (phob/phil‐9D‐74°) at 0.6 V for 16 h to investigate the effect of punching and reassembly of JPIP GDLs over longer‐term cell operation conditions (Figure 8f). No issues of stability arise within this timeframe, and in fact the current density of the JPIP GDL undergoes less fluctuation compared with the conventional hydrophobic GDL, although more stringent durability protocols will be carried out in the future to identify any longer‐term trends in performance. The results confirm the robustness of the JPIP GDLs and suggest that friction‐locking and MPL coating is sufficient to maintain the physical integrity during operation. This is not surprising, since MEAs are placed under significant lateral mechanical compression, confining the JPIP GDLs within the cell holder.^[^ ^57^, ^58^ ^]^ Furthermore, after disassembly of the cell, the JPIP GDLs remained intact according to visual inspection.

## 4 Discussion

The above results clearly show i) that incorporating hydrophilic pathways into the GDL significantly improves PEFC performance; ii) that the area ratio or number of hydrophilic pathways is critically important, and iii) that the relative hydrophilicity of pathways should also be considered. The general improvement in performance is attributed to the relative difference in capillary pressure between the hydrophobic and hydrophilic regions of the GDL. This drives a flux of water from the hydrophobic domains toward the hydrophilic domains. As the number of hydrophilic disks in the GDL increases, the total interfacial contact area between the hydrophobic and hydrophilic domains increases correspondingly, maximizing the rate of water transport and mitigating the accumulation of water at high current density. In future work, this will be investigated in more detail by further varying the shape, size, and spacings of the hydrophilic regions to control the interfacial contact area more precisely.

Meanwhile, as the water contact angle of the hydrophilic GDL disks decreases, the difference in capillary pressure between the hydrophobic and hydrophilic pathways also varies correspondingly. For phob/phil‐9D‐116, the difference is small, limiting the flux of water from the hydrophobic to hydrophilic regions. For phob/phil‐9D‐74, the difference in capillary pressure is larger, increasing the water flux into the hydrophilic pathways and thus significantly improving performance. We speculate that the decrease in performance for phob/phil‐9D‐0 is due to the high hydrophilicity of these domains relative to the ribs of the graphite flow field (WCA = 97°, Figure S9, Supporting Information), resulting in accumulation of water at the interface until the breakthrough pressure is reached, allowing the water to drain into the channel. Moving forward, it will be important to characterise the distribution of accumulated water in the cell via techniques such as neutron scattering and X‐ray computed tomography, as well as carefully optimising the relative hydrophilicity of the different components in the system.

Finally, the JPIP technique is highly amenable to scale‐up using existing industrial practices. The die‐cutting and pressing steps central to JPIP GDLs are compatible with roll‐to‐roll processing at commercial scale, whilst automated precision punching and robotic insertion of the hydrophilic disks into pre‐punched hydrophobic GDL sheets can rely on pick‐and‐place machinery already used in battery and fuel cell manufacturing today. Furthermore, hydrophilic GDL production via immersion in hydrogen peroxide can be integrated as a scalable and straightforward batch process or even incorporated as an inline process. Friction locking of the disks and partial bonding with the MPL ensure mechanical integrity without requiring additional adhesives or processing steps. Furthermore, the modular and geometric flexibility of JPIP GDLs will allow for programmable wettability patterning tailored to specific flow field designs or operating regimes, depending on the selected die. Together, these features make JPIP an industrially viable method for large‐area, high‐throughput production of patterned GDLs, supporting commercial‐scale deployment of high‐performance fuel cells.

## 5 Summary and Conclusion

In this work, Jigsaw Puzzle Inspired Patterning (JPIP) was presented as a new method to introduce precisely patterned hydrophilic pathways into hydrophobic GDLs and control the capillary pressure. Precise holes were punched in conventional hydrophobic GDLs whilst, disks of corresponding size were punched out of a hydrophilic GDL (prepared by chemical treatment in hydrogen peroxide). The hydrophilic discs were pressed into the corresponding holes of the hydrophobic GDL matrix and fixed by friction locking to create a hybrid GDL with patterned hydrophilicity. The hybrid GDLs were then coated with an MPL and incorporated into PEFCs. In all cases, the JPIP GDLs outperformed a conventional unmodified GDL. Increasing the number of hydrophilic domains resulted in improved PEFC performance due to lower oxygen transport resistance. Moreover, varying the hydrophilicity of the inserted disks had a significant effect on PEFC performance, with an intermediate water contact angle of 74° being optimal in this case. Decreasing the water contact angle further resulted in a decrease in performance attributed to water accumulation near the flow field ribs. Overall, this work introduces a new and unique method to create complex and precise hydrophilic patterns within a hydrophobic GDL structure, resulting in a clear enhancement in PEFC performance.

## Conflict of Interest

The authors declare no conflict of interest.

## Supporting information



Supporting Information

## Data Availability

The data that support the findings of this study are available from the corresponding author upon reasonable request.
