# Supplementary material for: Jigsaw Puzzle Inspired Patterning of Gas Diffusion Layers for Enhanced Water Management in Polymer Electrolyte Fuel Cells
Source: Adv Sci (Weinh). 2025 Sep 3;12(43):e07918. doi: 10.1002/advs.202507918 (PMC12631844; doi:10.1002/advs.202507918)
Supplement: Supplementary file 1 — Supporting Information [file ADVS-12-e07918-s001.pdf]

Jigsaw Puzzle Inspired Patterning of Gas Diffusion Layers for Enhanced Water Management in Polymer Electrolyte Fuel Cells

Enes Muhammet Can,[a,b,c,*] Masamichi Nishihara,[c,d] Kazunari Sasaki,[c,d,e] and Stephen Matthew Lyth[c,f,*]

a Mechanical Engineering Department, Faculty of Engineering and Natural Sciences, Bursa Technical University, Bursa, 16310, Türkiye

b Mechanical Engineering Department, Faculty of Engineering and Architecture, Kırşehir Ahi Evran University, Kırşehir, 40100, Türkiye

c Next-Generation Fuel Cell Research Center (NEXT-FC), Kyushu University, 744 Motooka, Nishi-ku, Fukuoka, 819-0395, Japan

d International Research Center for Hydrogen Energy, Kyushu University, 744 Motooka, Nishi-ku, Fukuoka, 819-0395, Japan

e Department of Hydrogen Energy Systems, Graduate School of Engineering, Kyushu University, 744 Motooka, Nishi-ku, Fukuoka, 819-0395, Japan

f Strathclyde Incubator for Green Hydrogen Technologies (SigH_2t), Department of Chemical and Process Engineering, University of Strathclyde, Glasgow G1 1XL, United Kingdom

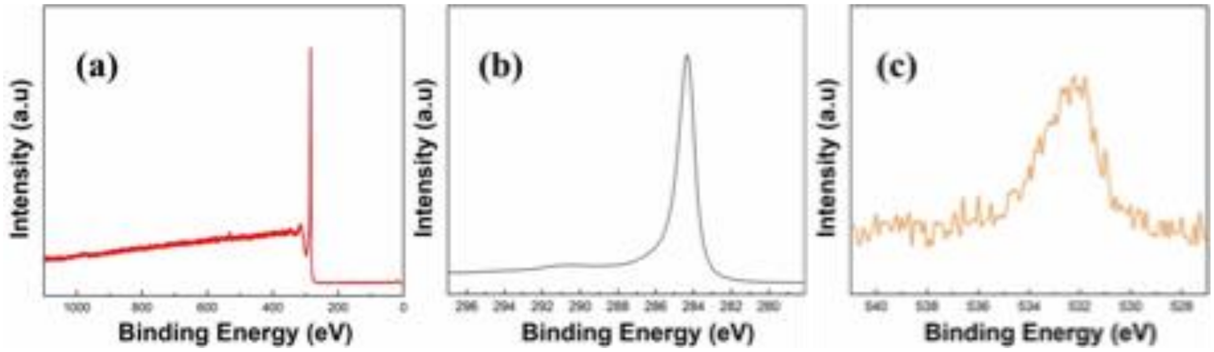

Figure S1. XPS spectra of SGL 29AA: (a) survey scan; (b) C 1s; and (c) O 1s region.

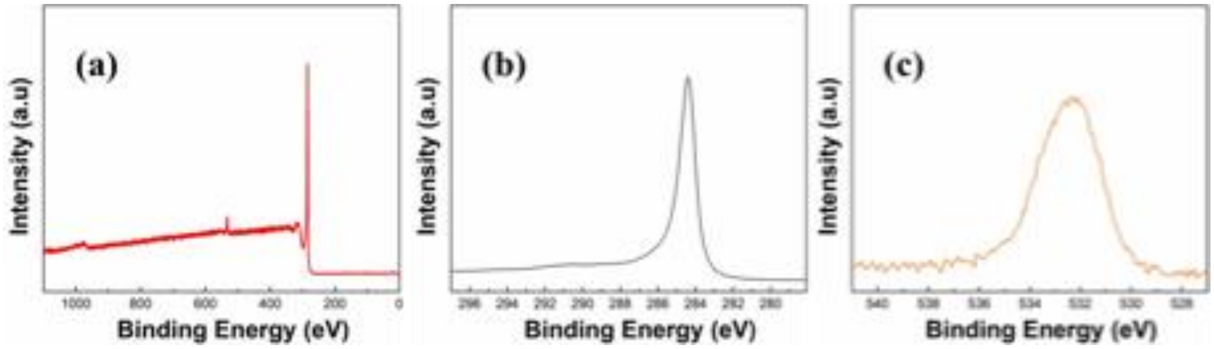

Figure S2. XPS spectra of SGL 29AA after hydrogen peroxide treatment for 12 hours: (a) survey scan; (b) C 1s, and (c) O 1s region.

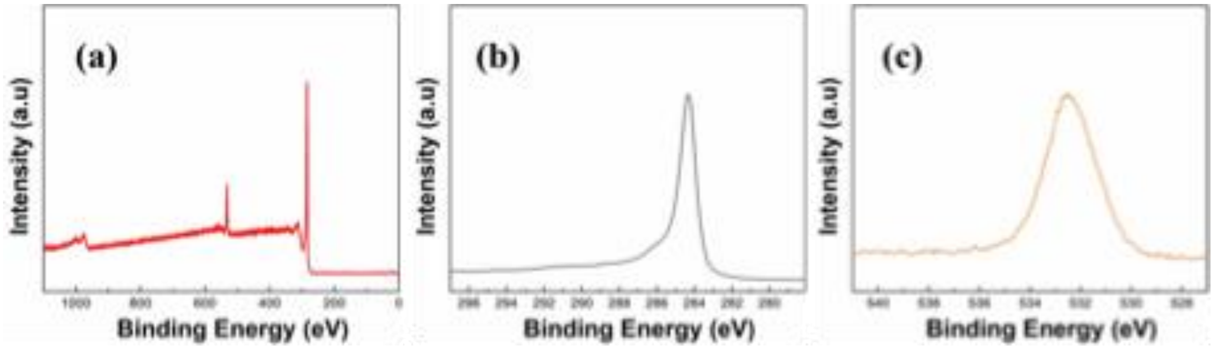

Figure S3. XPS spectra of SGL 29AA after hydrogen peroxide treatment for 24 hours: (a) survey scan; (b) C 1s, and (c) O 1s region.

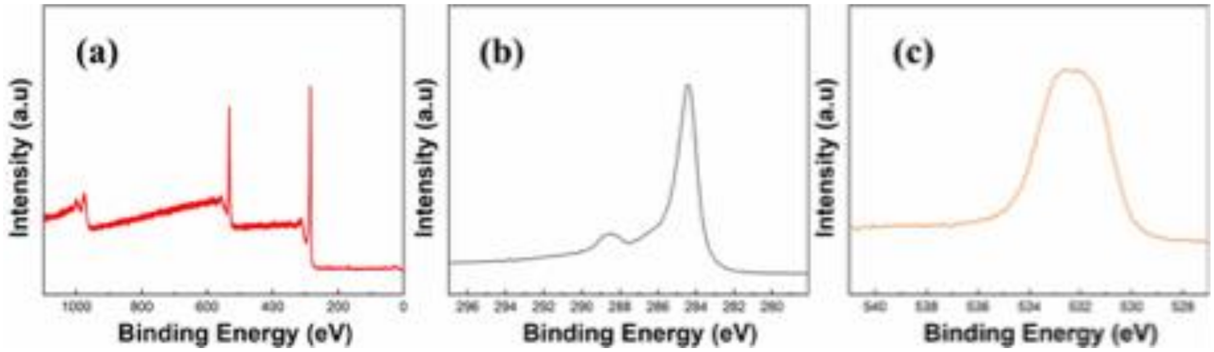

Figure S4. XPS spectra of SGL 29AA after hydrogen peroxide treatment for 48 hours: (a) survey scan; (b) C 1s, and (c) O 1s region.

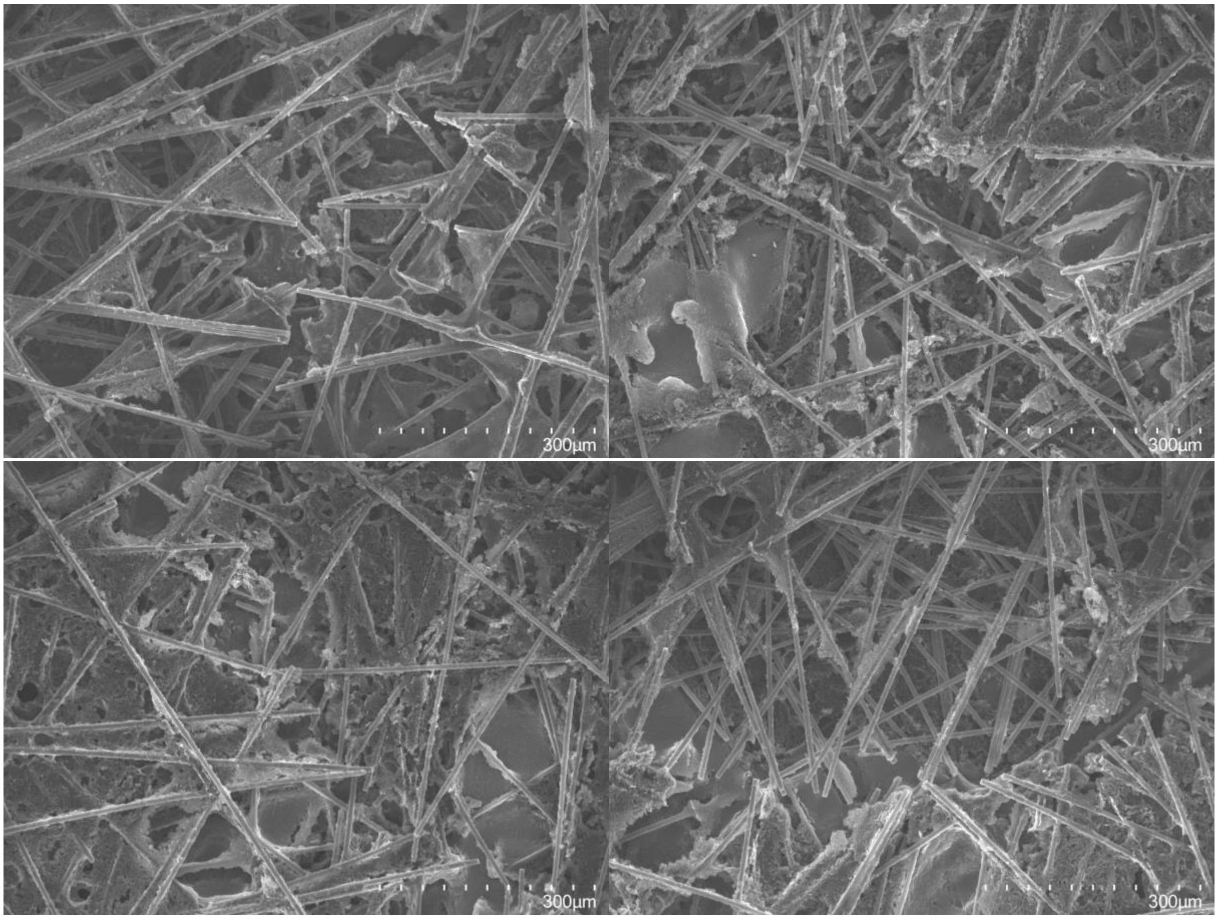

Figure S5. Representative SEM images of the JPIP GDL surface after re-assembly.

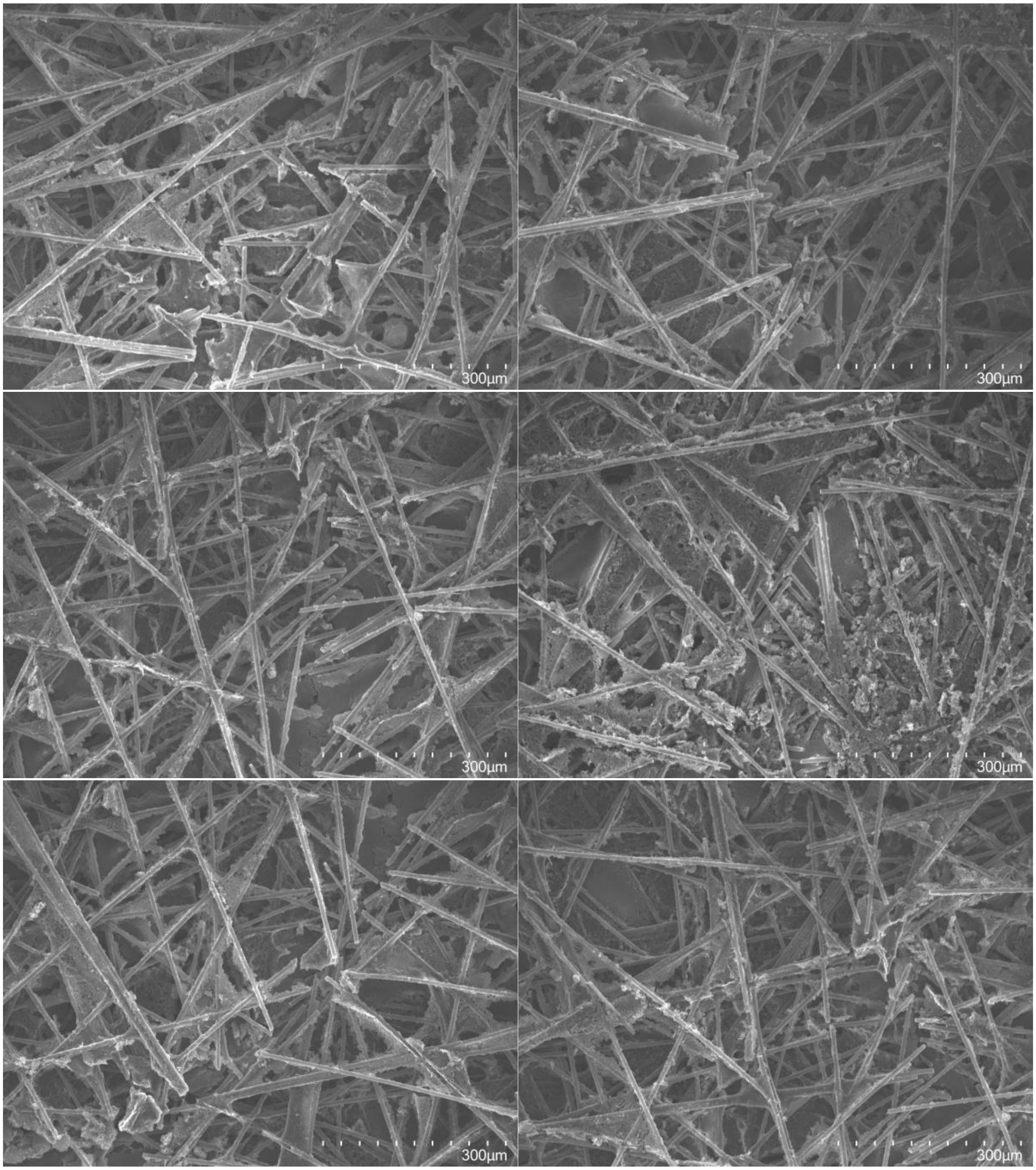

Figure S5 (cont.). Representative SEM images of the JPIP GDL surface after re-assembly.

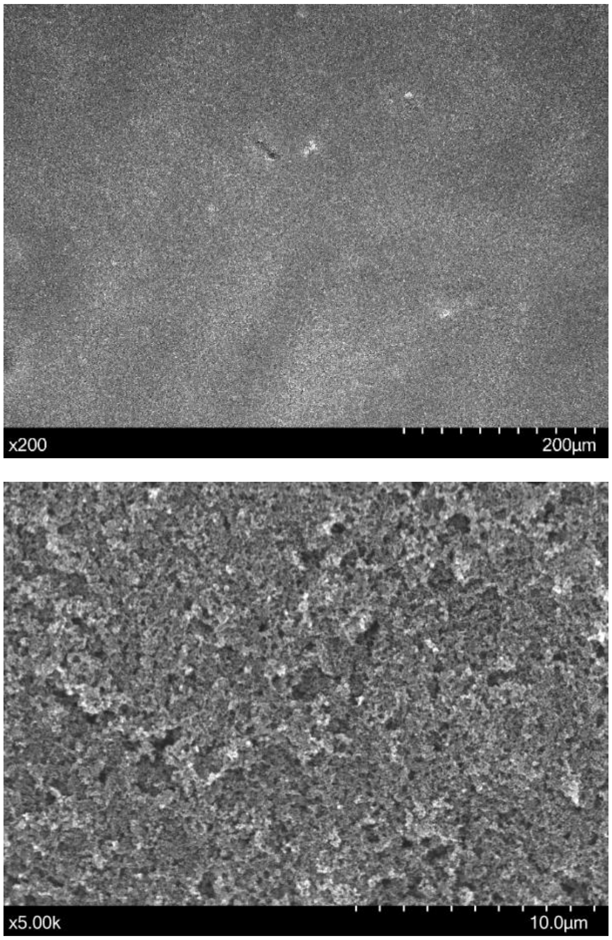

Figure S6. Representative SEM images of MPL surface coated onto a JPIP GDL.

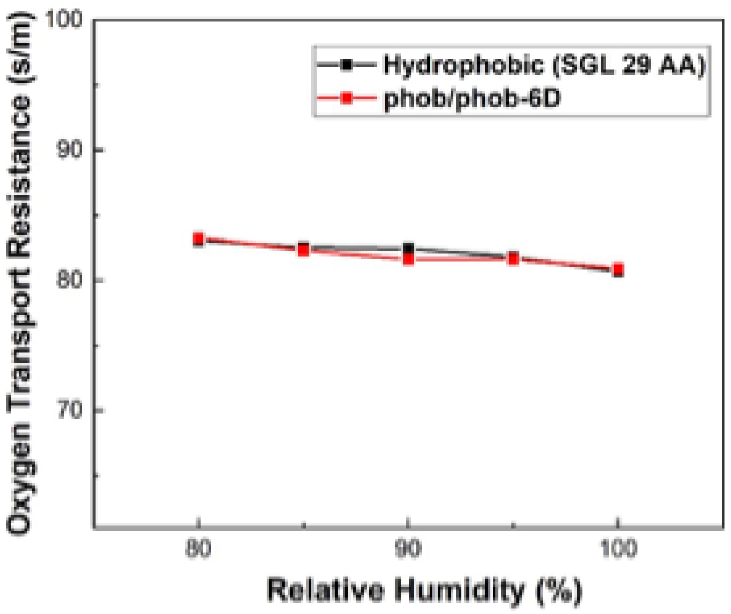

Figure S7. Oxygen transport resistance results for a conventional hydrophobic GDL (SGL 29 AA) and for a JPIP GDL phob/phob-6D.

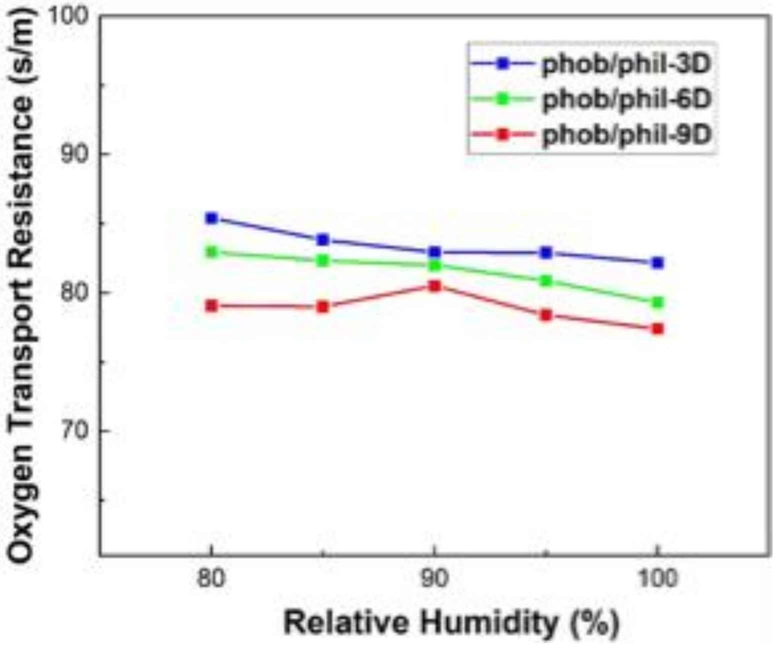

Figure S8. Oxygen transport resistance results of conventional hydrophobic GDL and phob/phil-3D, phob/phil-6D, phob/phil-9D.

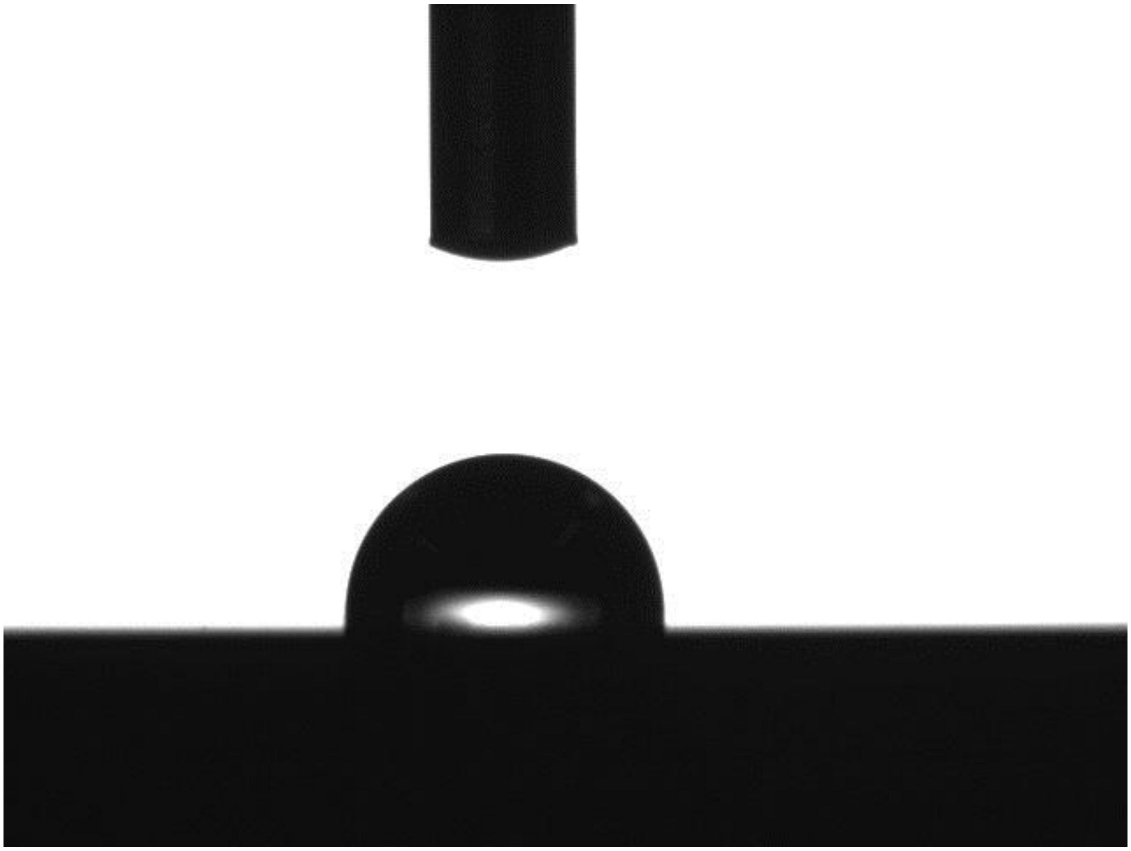

Figure SI 9. Water contact angle measurement of the rib of the flow channel (measured as 97 º).
